# Phylodynamic inference suggests introductions as main driver of Mpox Clade II outbreak in 2022 in Slovenia

**DOI:** 10.1017/S0950268825100587

**Published:** 2025-09-19

**Authors:** Bastiaan Van der Roest, Egil A. J. Fischer, Don Klinkenberg, Martin C. J. Bootsma, Mojca Maticic, Katarina Resman-Rus, Miša Korva, Tatjana Avsic-Zupanc, Mirjam Kretzschmar

**Affiliations:** 1 Julius Center for Health Sciences and Primary Care, University Medical Center Utrecht, Utrecht University, Utrecht, The Netherlands; 2 Department of Population Health Sciences, Faculty of Veterinary Medicine, Utrecht University, Utrecht, The Netherlands; 3 Center Epidemiology and Surveillance of Infectious Diseases, National Institute for Public Health and the Environment (RIVM), Bilthoven, The Netherlands; 4 Department of Mathematics, Faculty of Science, Utrecht University, Utrecht, The Netherlands; 5 Centre for Complex System Studies (CCSS), Utrecht University, Utrecht, The Netherlands; 6 Faculty of Medicine, University of Ljubljana, Ljubljana, Slovenia; 7 Clinic for Infectious Diseases and Febrile Illnesses, University Medical Centre Ljubljana, Ljubljana, Slovenia; 8 Institute of Microbiology and Immunology, Faculty of Medicine, University of Ljubljana, Ljubljana, Slovenia

**Keywords:** Mpox, phylodynamic modelling, surveillance, transmission inference, whole genome sequencing

## Abstract

In 2022, an Mpox clade II outbreak affected many countries. To optimize control, knowledge on the number of new introductions (human cases infected from outside the study population) versus local transmission is important. We extracted sequences of all 48 Mpox cases in Slovenia in 2022 from the NCBI database, of which 42 passed quality control. We estimated the number of introductions using the phylodynamic model phybreak by integrating genomic and epidemiological data and inferred transmission events. By repeating this analysis with weekly cumulative case data, we assessed if introductions could have been reliably inferred in real time. The number of introductions, estimated after the outbreak ended, was 19 (95% CI: 13–29), and two larger transmission clusters existed. As these introductions occurred throughout the outbreak, we conclude that the Slovenian Mpox outbreak was mainly driven by new introductions. Analysing the data ‘in real time’ would have only slightly overestimated the number of introductions per week, capturing the trend of introductions as main driver of the outbreak. This makes it useful for guiding control policy during outbreaks, prioritizing the rapid identification of cases among travellers, and with that preventing emergence of new transmission chains.

## Introduction

In 2022, the world faced an unexpected global outbreak of Mpox (formerly known as monkeypox), a rare viral disease typically found in Central and West Africa. Before 2022, Mpox cases outside of Africa were sporadic and associated with international travel or exposure to imported animals [1]. However, the outbreak that began in May 2022 quickly escalated, with more than 92,000 confirmed cases spread across more than 110 countries [2–4]. The majority of Mpox cases were found in the men who have sex with men (MSM) population. The monkeypox virus (MPXV) is genetically divided into two clades, of which Clade I and IIa are endemically circulating in animal reservoirs. Clade IIb has been continually circulating in humans since 2016, and in 2022, a new lineage B.1 emerged [5]. The widespread outbreak in 2022 was largely driven by this Clade II B.1 lineage, with significant human-to-human transmission, particularly through close contact [3, 6].

Decisions on how to intervene in an infectious disease outbreak hinge on understanding the factors driving the outbreak. In the case of a pandemic, there is potential for a significant and continuous influx of cases from outside a population. For example, for HIV-1, continuous and frequent cross-country transmission has been observed [7], and import is the main cause of *Mycobacterium tuberculosis* infections in low-incidence countries [8, 9]. Such outbreaks demand a focus on early detection in travellers and migrants, whereas in other instances the emphasis should be on the mitigation of transmission among residents. In the following, we will use the term ‘driven by introductions’ to describe an outbreak where this continuous influx plays a major role in the infection dynamics. In theory, for Mpox these introductions could either result from human dynamics, or from spill-over events from animal reservoirs. However, as the spread of the Mpox lineage B.1 in Europe occurs through human-to-human transmission, we use introductions here to indicate cross-country transmission through human dynamics. The use of epidemiological data, including data obtained through contact tracing, makes it possible to assess the role of introductions as the driving force of the outbreak. When such data are lacking, it becomes necessary to employ alternative methods to infer transmissions and introductions. When genomic data are available, phylodynamic inference could be done to infer who infected whom. Recently, the phylodynamic method phybreak was extended to distinguish introductions from transmission events [10].

In the period between May and September 2022, 129 suspected Mpox cases were identified in Slovenia, a central European country with a population of two million. Swabs of various skin lesions, anogenital swabs, or skin biopsies were analysed by the Laboratory for Diagnosis of Zoonoses at the Institute of Microbiology and Immunology, Faculty of Medicine, University of Ljubljana, of which 49 (38%) of these 129 cases were found positive by two different real-time polymerase chain reaction (RT-PCR) tests – LightMix^®^ Modular assays for Orthopoxvirus (OPV) and MPXV (Roche, TIB MolBiol, Germany). Of all positive cases, whole genome sequences were obtained by Resman Rus et al. [11]. They performed a phylogenetic analysis with 48, of the 49, high-quality sequences, on which related clades of cases were identified, indicating the potential for chains of transmission within the country. In the same analysis, at the peak of the outbreak in July 2022, four distinct genetic lineages were identified, indicating that there had been multiple introductions. As a consequence, it was unclear what the contribution of introduction and local transmission were to the outbreak. Phylodynamic inference makes this distinction on a finer resolution, making it possible to infer introductions even within clades.

In this study, we aimed to assess the extent to which the outbreak in Slovenia was driven by introductions or local transmission events. We performed a phylodynamic analysis combining the sequences of the 48 cases with epidemiological data, that is, detection times, and a model of transmission dynamics to infer transmission events and introductions. This inference provided insight into the extent to which the outbreak was driven by introductions. Furthermore, we examined the feasibility of investigating the contributions of introductions and local transmission by this method during the course of the outbreak, by comparing the numbers of identified introductions in real time with those numbers estimated in the analysis of the complete outbreak. Knowing in real time whether introduction or local transmission is the main driver enables a timely and focused response to the outbreak.

## Methods

### Data

A total of 48 MPXV sequences, as used in Resman Rus [11], were retrieved from the National Center for Biotechnology Information (NCBI) database with sampling dates between May and September 2022 in Slovenia. The accession numbers can be found in Supplementary Table S1. The sequences were aligned using Squirrel, and a maximum likelihood phylogenetic tree was constructed using IQTree2 [12]. A temporal signal was examined and the quality of the sequences was controlled using TempEst version 1.5.3 [13]. Outliers of a linear regression on the root-to-tip divergence were identified as follows. A sequence first was designated as outlier if its corresponding residual was more than 3 times the standard deviation of the mean residual. Of the remaining sequences, further outliers were identified if the branch length of the tips in the maximum likelihood phylogenetic tree (constructed with IQTree2) to their parent nodes were more than 30 times the median branch length of all tips. Doing so, sequences were identified as outliers because they had substantial longer branch lengths than expected. Outliers were excluded from further analyses, and the slope of the linear regression line was recalculated to estimate the mutation rate.

### Phylodynamic modelling

Inference of transmission events was conducted using the phylodynamic model incorporated into the phybreak package [14] written in the R computing language [15]. This model simultaneously estimates phylogenetic and transmission trees based on genetic sequences and their sampling times and additional epidemiological data, for example, the generation time interval. This methodology, in comparison to other transmission tree inference methods, also allows for the estimation of the expected number of introductions of a pathogen into a sampled population [10]. As Slovenia was one of the few countries with a sequence coverage of 100%, it was possible to conduct a phylodynamic analysis using phybreak, which requires a completely sampled population.

We used a normal distribution prior for the mean generation time interval, with a mean equal to the serial interval of 8.5 (SD: 3) days [16]. The mean sampling time interval had a prior normal distribution with a mean equal to the incubation time of 10 (SD: 3) days [16]. For analyses with a fixed mutation rate, we used a rate of 1.13 × 10^*−*4^ mutations/site/year [17]. For analyses in which the mutation rate was estimated, we used a normal distribution prior with a mean of 2 × 10^*−*4^ (SD: 0.5 × 10^*−*4^) SNPs/site/year based on the slope of the linear regression with TempEst. We ran chains of 100,000 cycles, with sampling occurring at 10-cycle intervals.

In conducting the sensitivity analysis with unobserved cases, we added either five (equivalent to 10% of all cases) or nine (equivalent to 20% of all cases) cases to the sampled cases. To ensure the absence of any information in the genetic data, all cases were assigned a sequence comprising solely *N*s. To reflect the epidemic in Slovenia, sampling times were drawn from a Gamma distribution with a mean of 60 days, from the first detection date at May 23, and a shape of 3, comprising ten sets for both the five and nine unobserved cases (Supplementary Figure S1).

The estimated number of introductions is calculated as the sum of the supports of all cases that are identified as index cases in the inference. Clusters were derived on a per MCMC cycle basis, and an average distribution of cluster sizes is presented.

### Real-time inference

To perform the real-time inference, the data were divided into 16 weekly segments, starting from the date of the initial sample (Supplementary Figure S2). On a weekly basis, transmissions were inferred with phybreak using the sequences sampled up to that point in time. The estimated number of introductions was then compared to the results of the retrospective analysis of the complete outbreak. A sensitivity analysis of the real-time inference was conducted by introducing a 1-week waiting period before analysis. Cases sampled up to week *i* + 1 were employed to estimate the number of introductions in week *i*, given the possibility of sampling links between introductions in week *i* in week *i* + 1.

## Results

### Quality control of sequences

Before we could infer transmission events, we conducted quality control on the 48 Slovenian MPXV sequences. For samples MPXV-12, MPXV-15, MPXV-30, MPXV-38, MPXV-46, and MPXV-47, we found evidence for a greater number of unique single nucleotide polymorphisms (SNPs) than expected over time (Supplementary Figure S3), such that we excluded these samples from subsequent analyses. Exclusion means also that we do not count these cases in the number of introductions. In the remaining set of sequences, a maximum of 30 SNPs were observed between two sequences. Furthermore, the majority of distances were between the 0 and 5 SNPs (Supplementary Figure S4). No evidence was found for bad quality of the remaining sequences.

### Inference of the complete outbreak

**Figure 1.**
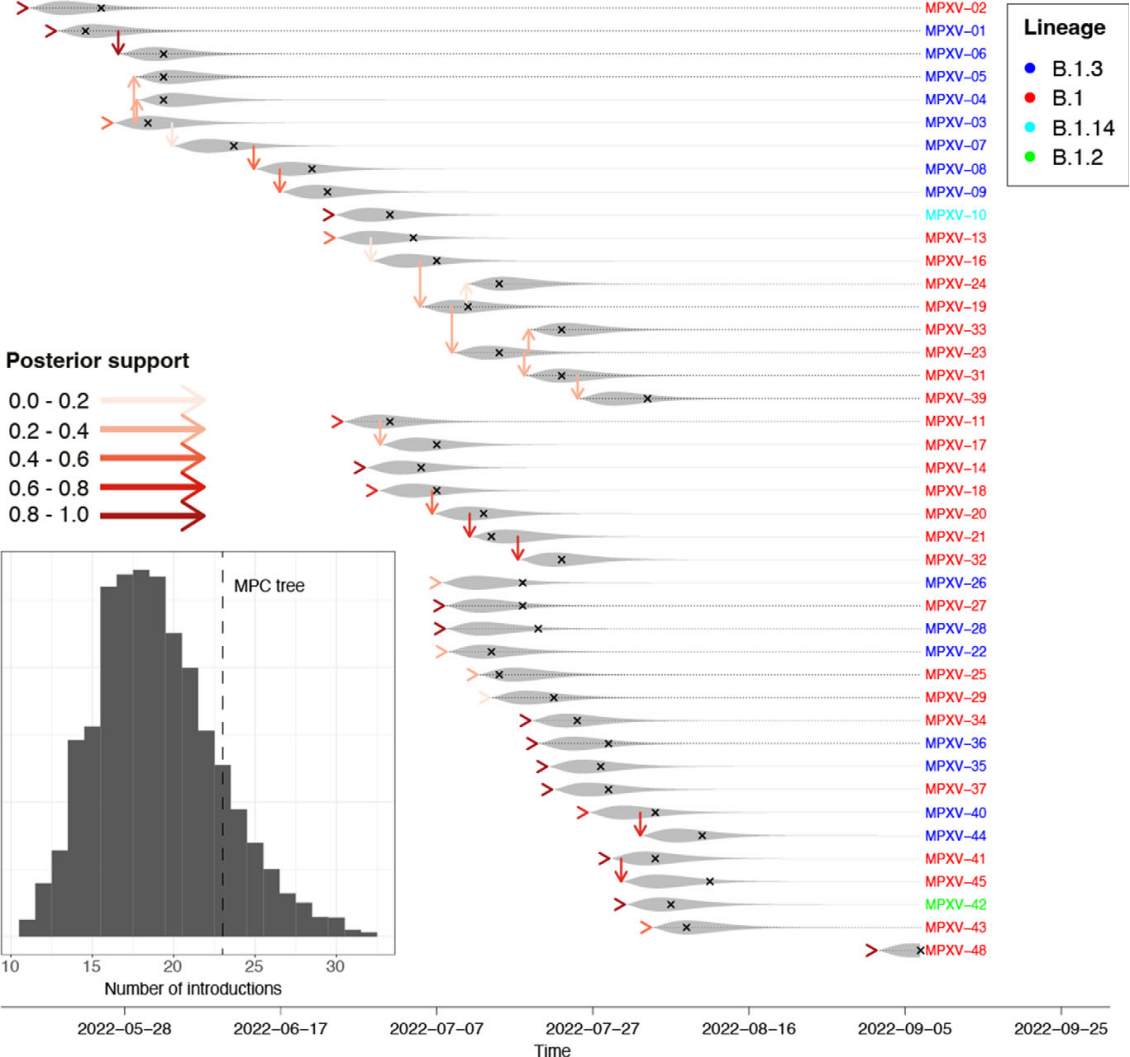
Maximum parent credibility (MPC) transmission tree of 2022 Slovenian MPXV outbreak. Each line represents a case, with grey shades indicating the infectiousness distribution of a case and crosses indicating the sampling times. Horizontal arrow heads indicate introductions and vertical arrows indicate transmission, with support coloured from white to red. Case IDs are coloured according to their MPXV lineage. In-lay: posterior distribution of number of introductions. The MPC tree has 23 introductions.

To infer transmission events, we employed the phylodynamic method phybreak with priors for the mean generation time (8.5 days) and mean sampling time (10 days). The substitution rate was estimated by the model. We inferred a number of 19 (posterior median, 95% CI: 13–29) introductions, with the majority of introductions occurring around the peak of the outbreak in July 2022. Figure 1 shows the maximum parent credibility tree, which is a realization of the outbreak with the most likely phylogenetic tree based on the sequences. Of the identified introductions, on average, 14.0 were unlinked cases, that is, cases without a transmission link to another case. Linked cases not only were often found as transmission pairs, but also two larger clusters were found (Supplementary Figure S5). These larger clusters both started at the beginning of the outbreak, one consisting completely of lineage type B.1 and the other of B.1.3.

When the six removed cases, whose sequences had bad quality, were included without genetic information, we found a number of 21 (posterior median, 95% CI: 15–32) introductions. Cases MPXV-46 and MPXV-47 were unlinked cases with a support of, respectively, 87% and 85%, explaining the increase in median number of introductions. Cases MPXV-12, MPXV-15, MPXV-30, and MPXV-38 were infected by other cases, but were not found to connect transmission chains. We conclude that our results were not sensitive to our decision to remove these cases from the data.

A second sensitivity analysis was conducted to see whether index cases were infected by unobserved cases serving as intermediates between transmission chains. In clinical studies, it was found that between 1.3% and 6.5% of MPXV-infected individuals exhibited no symptoms [18–20]. Therefore, we included 10% and 20% (amounting to 5 or 9 cases) of hypothetical unobserved cases into the analysis, for which a sampling date was assumed, but no genetic information was available. For both scenarios, we simulated 10 outbreaks and inferred transmissions. We found that half of the unobserved cases were identified as being infected by observed cases, and respectively, 72% (2/2.8) and 82% (4.0/4.8) of these unobserved cases were identified as transmitting infection to observed cases (Table 1). Although unobserved cases were used as links between observed cases, the median number of introductions was in the 95% credibility interval of the inference without unobserved cases. So, we concluded that a small fraction of cases remaining unobserved will not affect the estimated contribution of introductions as main driver of the outbreak.

**Table 1.** Number of unobserved cases infected by observed cases, and how many of these infected unobserved cases transmitted to observed cases. Average median number of introductions is over 10 outbreaks

Number of unobserved cases	Infected by observed cases	Transmitted to observed cases	Average median number of introductions (95% CI)
5	2.8	2.0	18.3 (15.4–21)
9	4.8	4.0	17.9 (11.2–22.6)

Furthermore, with an increasing number of sequences available, the precision of the estimate for the mutation rate will increase. So, we assessed the effect of estimating the mutation rate based on the outbreak sequences. For this, we inferred a transmission tree of the complete outbreak, utilizing a fixed mutation rate of 1.13 × 10^*−*4^ SNPs/site/year, as determined in a phylogenetic analysis of 547 B.1 sequences [17]. This resulted in a median number of 23 (95% CI: 16–32) introductions, which was higher than the median of 19 introductions found when estimating the mutation rate (Supplementary Figure S6). Estimation of the mutation rate could therefore be employed without affecting the identification of the outbreak’s driving force. This allows for analyses performed in the beginning phase of an outbreak of a new pathogen, when there is not yet a large cohort-based mutation rate estimation.

### Track introductions over time

**Figure 2.**
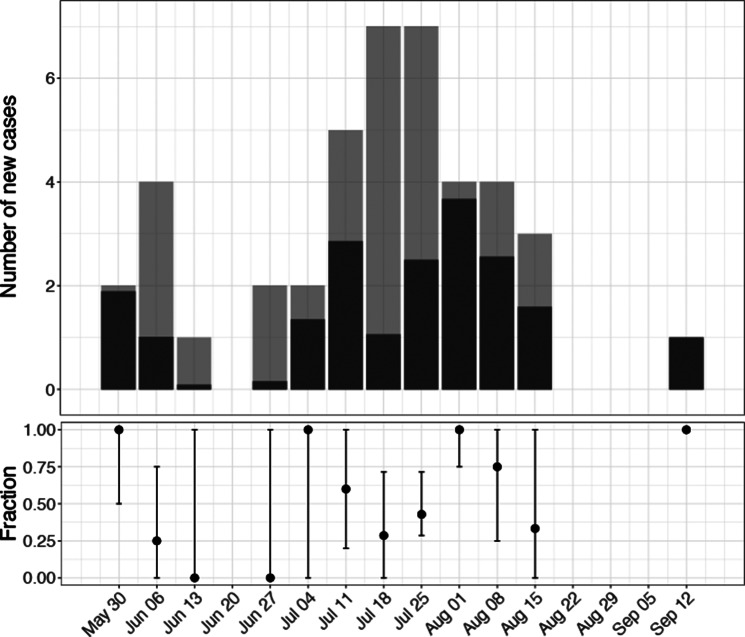
The estimated number of new cases attributed to introductions. Top: absolute number of cases. Total bar length gives the total number of new cases in a week (no bar indicates no cases), with the posterior mean number of cases attributed to introductions in black. Bottom: fraction of new cases attributed to introductions (dot is posterior median, line indicates 95% credible interval).

For the outbreak being driven by introductions, we wanted to know whether there was a continuous influx of new cases, resulting in small transmission chains. So we retrospectively tracked the number of introductions over time. Following the initial introductions in the first week, cases were infected by local spread, as observed by the decline in the fraction of introductions (Figure 2). However, during the peak of the outbreak, from July 11 till August 15, the fraction of new cases attributed to introductions increased again, reaching an average of 51%. After August 15, no new cases were identified till the last case, MPXV-48, at September 7, which was assigned as a new introduction. Therefore, we concluded that there was a continuous influx of new cases which likely drove the ongoing outbreak.

### Real-time inference

**Figure 3.**
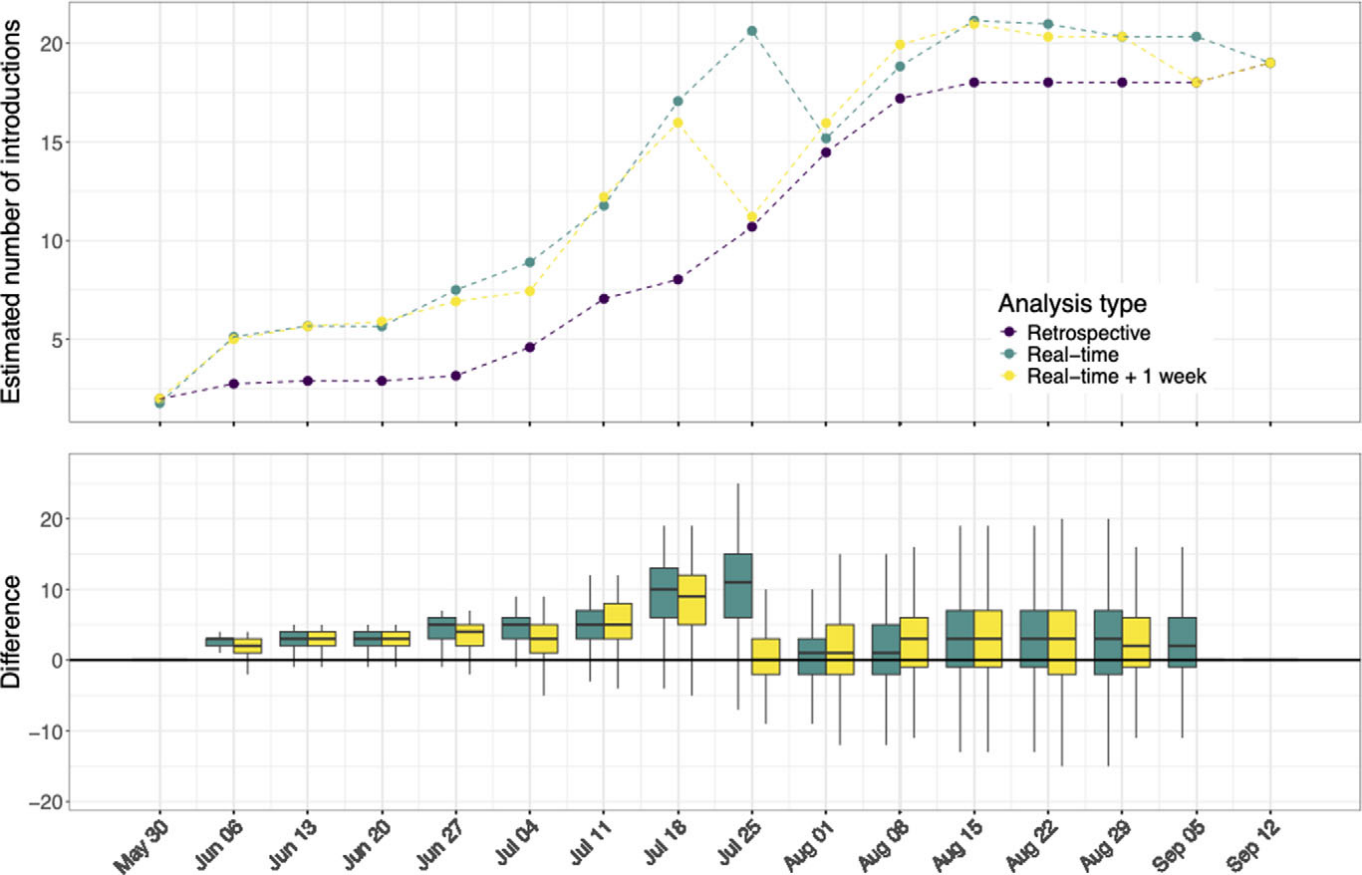
Weekly estimated number of introductions. Top: Traces of estimated number of introductions for the retrospective analysis (purple), real-time analysis (green), and real-time analysis with a 1-week waiting period (yellow). Bottom: Differences in number of introductions compared to the retrospective results.

To make timely informed decisions about public health control measures, it is vital to understand the driving force behind the outbreak while it is developing. We assessed the performance of phylodynamic modelling for real-time analyses, using the phybreak method. At the beginning of an outbreak, there is often limited knowledge about the transmission dynamics of a new pathogen. However, the posterior distributions of these parameters found by inference of the outbreak in the early weeks are in accordance with the given priors described in the methods (Supplementary Figure S1). The real-time inference estimated the number of introductions higher in all weeks compared to the retrospective analysis (Figure 3). On the contrary, the posterior mutation rate was lower till August 1. From that week, the mutation rate was comparable to the rate estimated in the retrospective analysis.

What makes analyses in real time more uncertain is the fact that a case detected in week *i* can have been infected by a case who is only detected in week *i* + 1. When we employ a 1-week waiting period, that is, number of introductions are estimated with cases sampled up to week *i*, while using all data collected up to week *i* + 1, the resulting estimates closely align with those derived without a waiting period.

**Figure 4.**
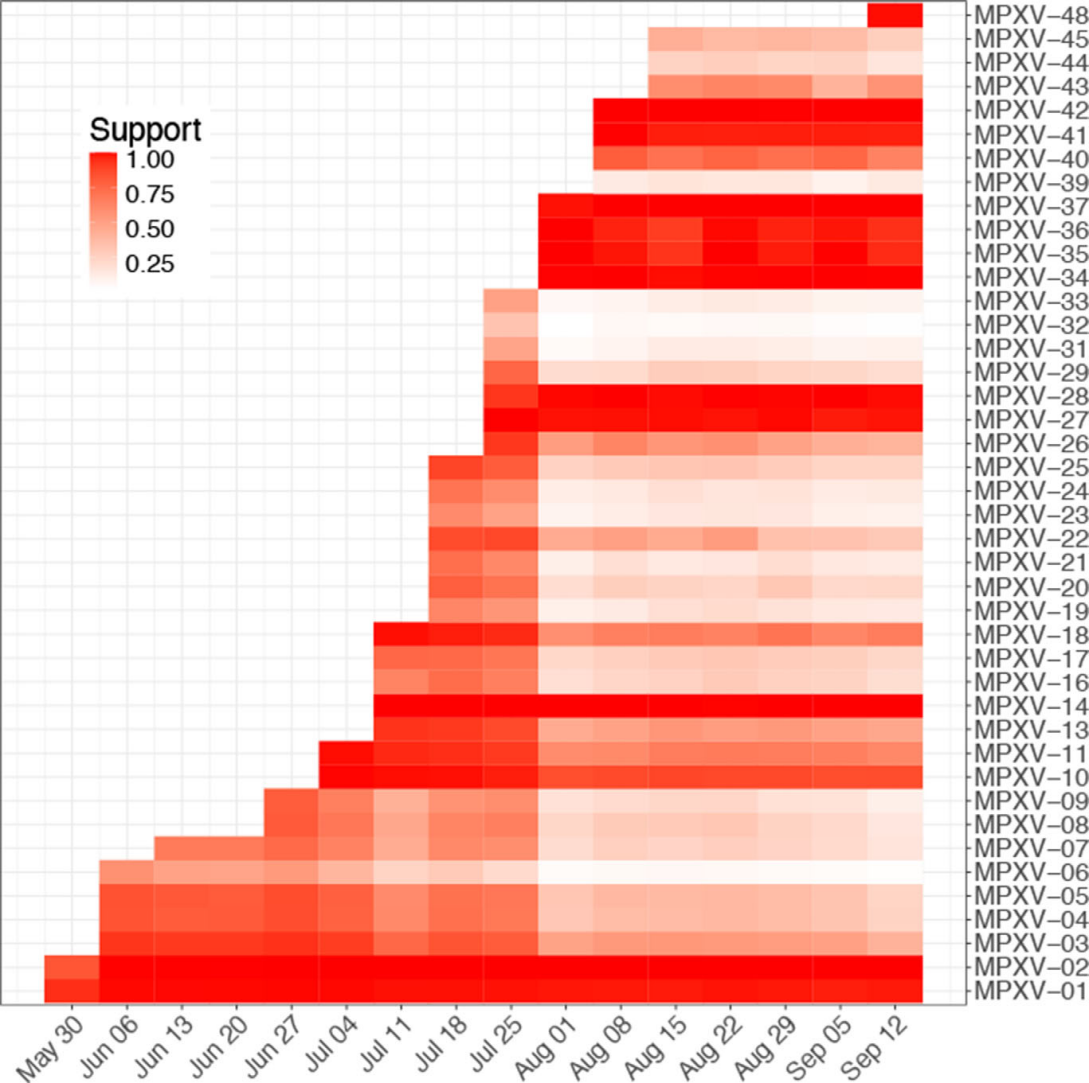
Posterior support for being an index. At weekly time points, a transmission inference was performed with the cases sampled up to that week.

Although the number of introductions was initially overestimated by the real-time inference, index cases with high support on September 12 were already found with similarly high support in the weeks that they were sampled (Figure 4). The overall support of index cases declined markedly on August 1, which is reflected in the decline in the estimated number of introductions on this date (Figure 3). However, there were no changes in which were the most likely index cases.

## Discussion

Understanding the drivers of outbreaks is crucial for guiding public health measures. In this study, we used a phylodynamic method to investigate the main driver of the 2022 Mpox outbreak in Slovenia.

We found a median of 19 introductions among the 42 cases included (Figure 1). Of these introductions, 74% (14/19) were unlinked cases, and support for two larger transmission clusters was found. At the peak of the outbreak, approximately half of all new cases were attributable to introductions. Importantly, adding unobserved cases had little impact on the number of introductions (Table 1), suggesting that the high number of introductions was not caused by missing links in transmission chains. These findings indicate that introductions were the main driver of the outbreak. Although real-time inference overestimated the number of introductions (Figure 3), the index cases with high support consistently remained index cases. This robustness suggests that such phylodynamic insights can guide public health decisions during ongoing outbreaks.

In many countries affected by the Mpox outbreak, multiple introductions from abroad were reported, often linked to international super-spreading events such as the LGBTQI+ pride on Gran Canaria, Spain [21–27]. These introductions were followed by localized spread, which dominated the outbreak’s progression in most regions. Western European countries reported 1.5–15% of the new cases being introductions after the first dissemination [25, 28]. However, in Slovenia, 51% of cases during the peak in mid-July were attributed to introductions. The rapid spread from late May to mid-July, and only occasional cases detected later in the summer, suggests that the infection did not penetrate dense local sexual networks as it did in other European regions [29, 30]. This limited spread and rapid containment of the outbreak likely reflect Slovenia’s prompt public health response including not only the public health control measures, but also activation of the MSM community to target prevention of further viral transmissions [31]. A comprehensive national communication strategy was implemented in collaboration with the largest LGBTQI+ civil society organization which played a significant role in raising awareness within this high-risk population. Its response rapidly followed the introduction of the interventions by the healthcare system, and remained persistently active throughout the whole outbreak period and even afterwards by promoting Mpox vaccination.

Incorporating epidemiological data, such as contact or travel histories, could enhance transmission tree accuracy.

However, such data were very limited. By means of interviews with managing doctors and retrospectively reviewing the medical documentation, we found that MPXV-10 had attended a foreign gay festival in the 3 weeks before detection. This confirmed the high support for this individual being an index case in the transmission tree. Moreover, MXPV-44 was found to have had sexual contact with a confirmed Mpox case in the last 3 weeks, which agrees with the strong indication of this individual having been infected by MPXV-40. This agreement between the available data and our inference underscores the method’s validity.

Phylodynamic modelling provides a powerful tool for real-time epidemiological analysis, but it has limitations. A complete dataset of sequenced cases is critical to avoid overestimation of introductions or mutation rates. Missing cases can distort results, emphasizing the need for comprehensive sampling during outbreaks. To overcome these limitations, it is optimal to sequence all positive identified Mpox cases and perform contact tracing in a standardized way. Knowledge about which positive cases had contact with each other, or which cases visited foreign countries, enhance the support of the inferred introductions or transmission events.

Estimation of the mutation rate during an outbreak is also challenging due to limited data. Early in the Mpox outbreak, estimates of mutation rates were influenced by sequence quality and quantity. Cases with more SNPs than expected, such as MPXV-34, MPXV-35, and MPXV-37 (Supplementary Figure S1), led to overestimated mutation rates and a subsequent decline in inferred introductions. Using a lower, fixed mutation rate derived from 547 sequences resulted in a higher median number of introductions, further supporting introductions as main driver of the outbreak.

Beyond the mutation rate, also other factors might have influenced estimation of the number of introductions. In phybreak, it is assumed that contact rates are equal between all individuals. If contact rates are more heterogeneous, it may affect the distribution of generation times and therefore inference of introductions. Also, change in social behaviour during the outbreak, for example, people decreasing their numbers of contacts, might change inference of introductions. More research is needed to assess the effect of contact rate heterogeneity in inferring the number of introductions.

## Conclusion

While most of the 25,000 Mpox cases in Europe in 2022 resulted from local transmission [28], our analysis revealed that a large proportion of infections in Slovenia were introduction events from other countries. We reached this conclusion through a retrospective analysis, with sensitivity analyses of model parameters and missing cases. Furthermore, we showed that the same conclusions could have been found in real time. By integrating phylodynamic modelling into outbreak surveillance, policymakers can obtain valuable insights to guide prevention and control strategies effectively. In small outbreaks with continuous occurrence of new introductions, we propose to prioritize rapid case finding in travellers and targeted risk-group communication to prevent infection when going abroad.

## Supporting information

10.1017/S0950268825100587.sm001 Van der Roest et al. supplementary material Van der Roest et al. supplementary material

## Data Availability

The sequence data and sampling meta-data (collection dates, host identifiers) are available via the National Center for Biotechnology Information (NCBI) database. The accession numbers can be found in Supplementary Table S1.

## Supplementary material

The supplementary material for this article can be found at http://doi.org/10.1017/S0950268825100587.

## Reproducibility statement

All analyses in this study were performed using the R package phybreak for Bayesian inference of transmission trees based on pathogen sequence data and sampling times. The following resources have been made publicly available to ensure full reproducibility:

- Code: All R scripts used for data pre-processing, model fitting, posterior sampling, and visualization are available at https://github.com/bastiaanvdroest/phybreak, along with clear instructions in the README file.
- Data: The sequence data and sampling meta-data (collection dates, host identifiers) are available via the National Center for Biotechnology Information (NCBI) database. The accession numbers can be found in Supplementary Table S1.
- Model settings: All priors, MCMC settings (e.g., number of iterations, thinning, burn-in), and parameter choices (e.g., generation interval, sampling delay distributions) used in phybreak are documented in the Methods section. Unless mentioned otherwise, the default parameter value is used.

We encourage others to re-use and adapt our code and methodology for similar analyses in molecular epidemiology and outbreak reconstruction.
