# Supplementary figures and images for: Phylodynamic inference suggests introductions as main driver of Mpox Clade II outbreak in 2022 in Slovenia
Source: Epidemiol Infect. 2025 Sep 19;153:e115. doi: 10.1017/S0950268825100587 (PMC12529423; doi:10.1017/S0950268825100587)

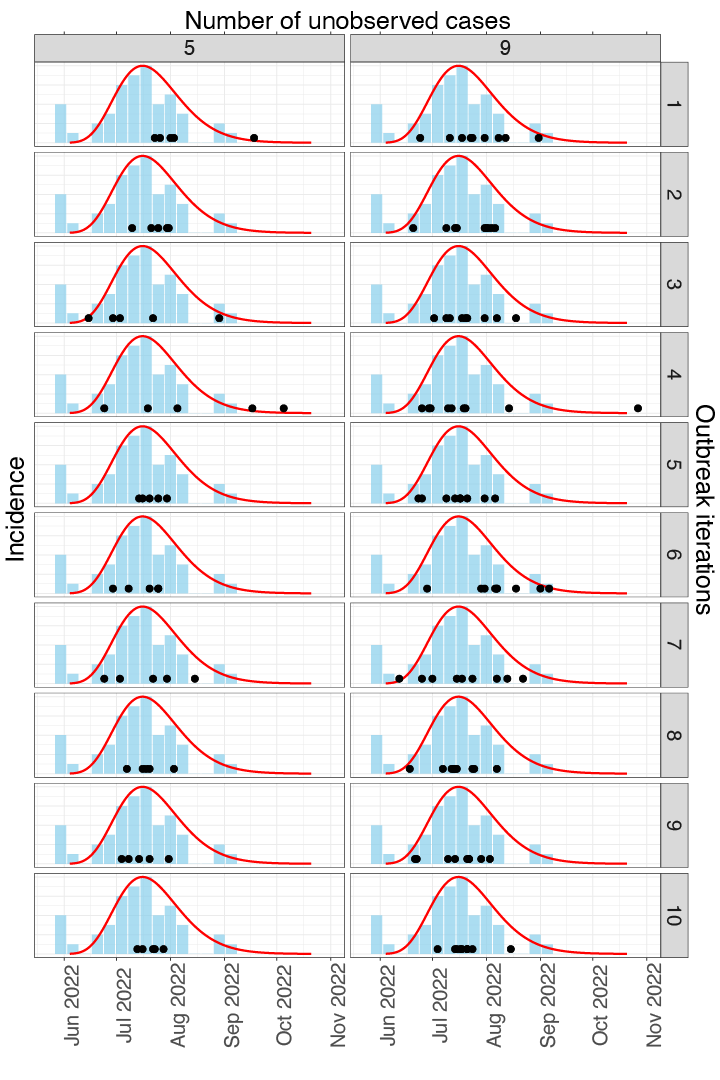

Supplement: Van der Roest et al. supplementary material [file S0950268825100587sup001.zip › S7_gamma_dist.png]

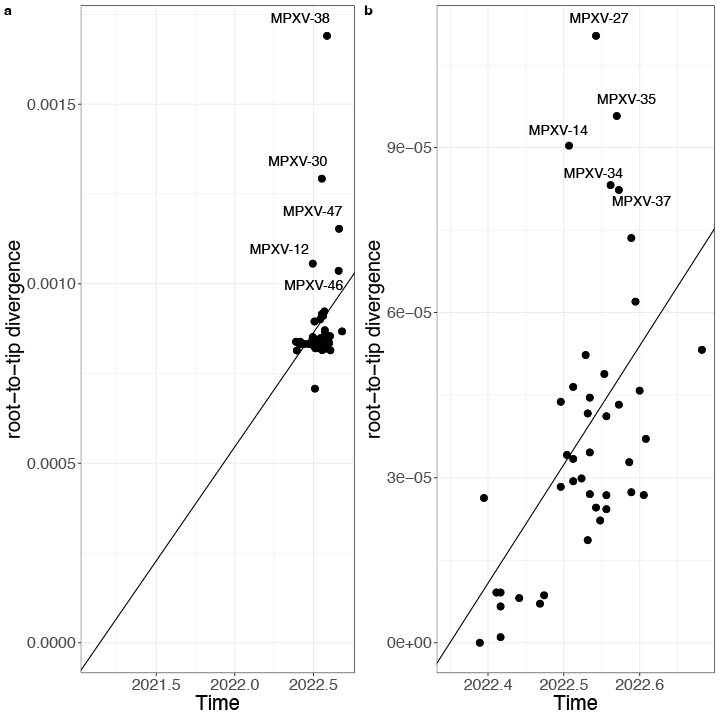

Supplement: Van der Roest et al. supplementary material [file S0950268825100587sup001.zip › S1_tempest_results.png]

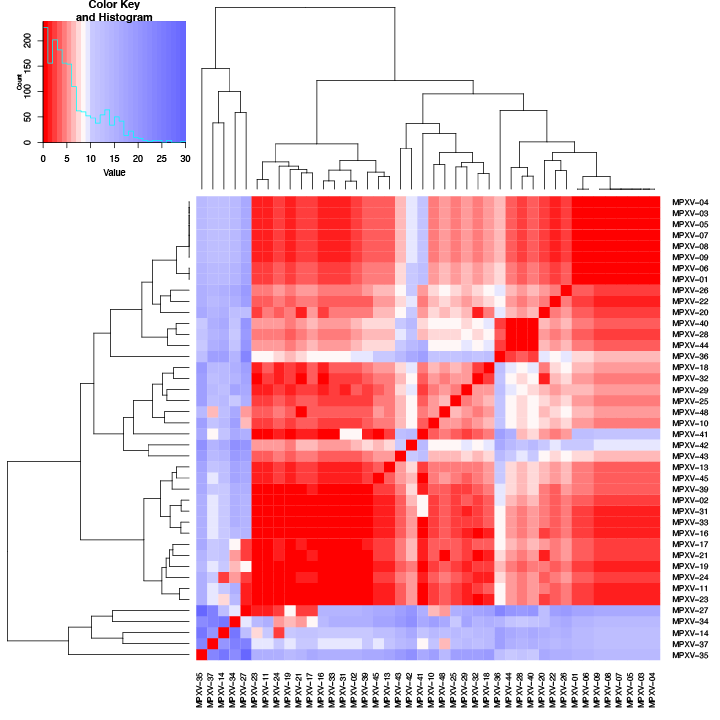

Supplement: Van der Roest et al. supplementary material [file S0950268825100587sup001.zip › S2_heatmap_SNPdist_removedoutliers.png]

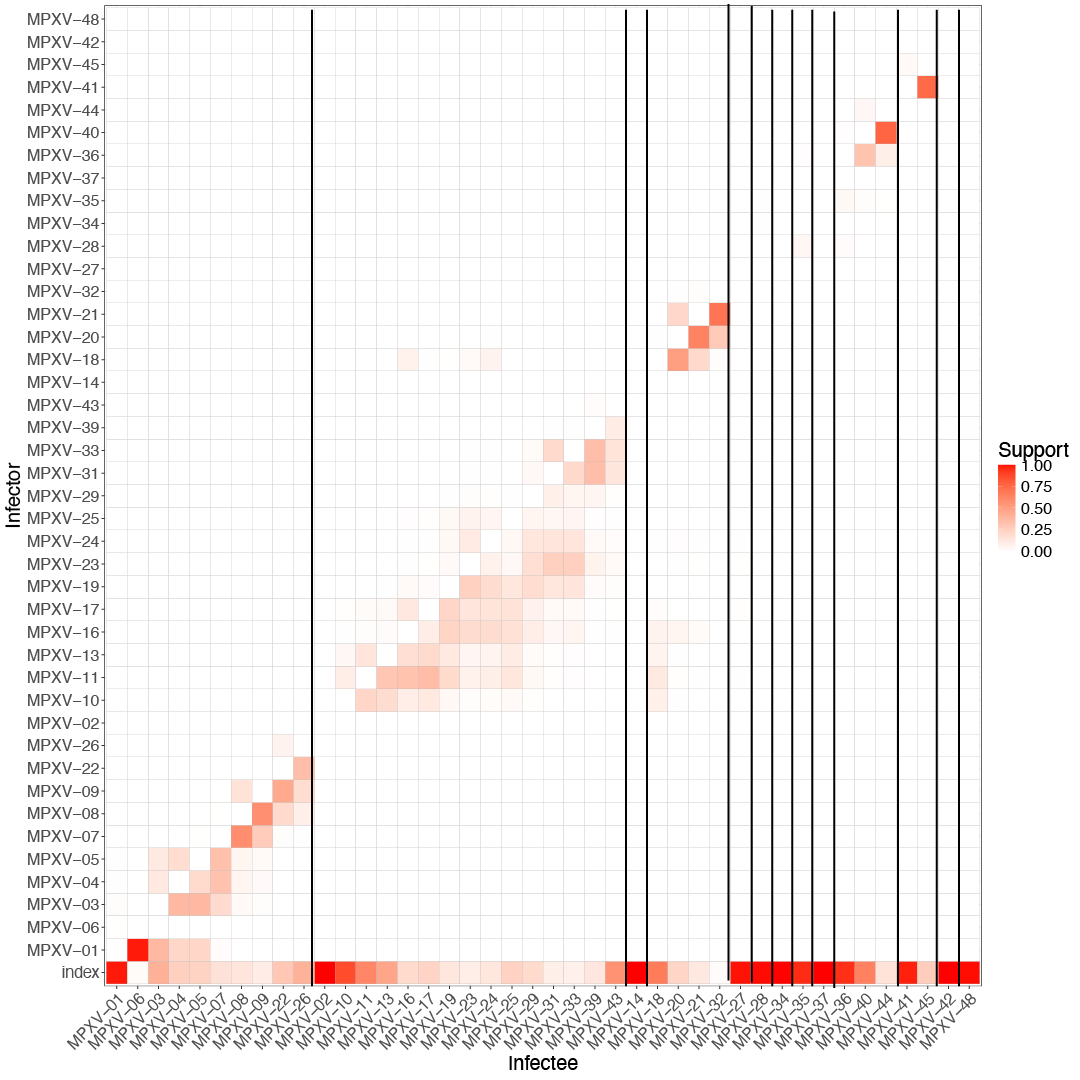

Supplement: Van der Roest et al. supplementary material [file S0950268825100587sup001.zip › S3_support__completeoutbreak_ordered.png]

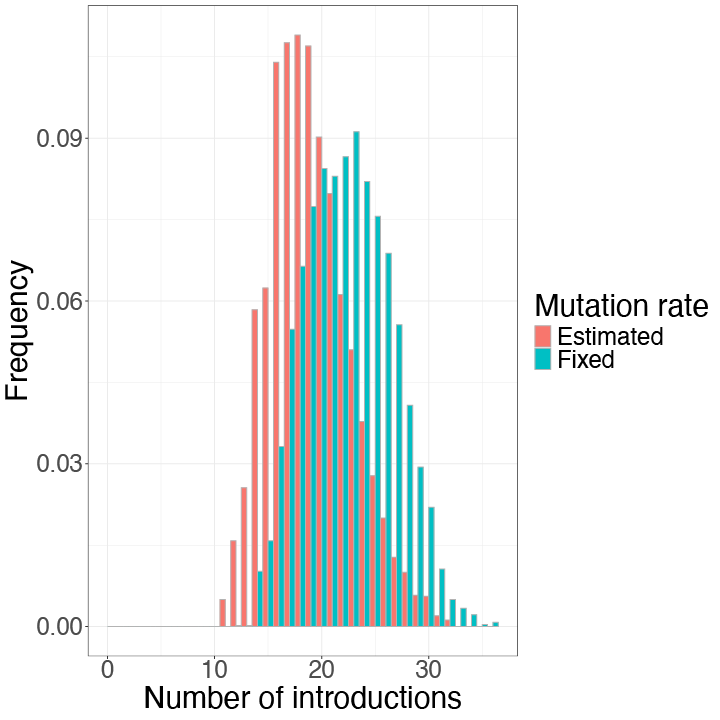

Supplement: Van der Roest et al. supplementary material [file S0950268825100587sup001.zip › S4_intro_est_fixed.png]

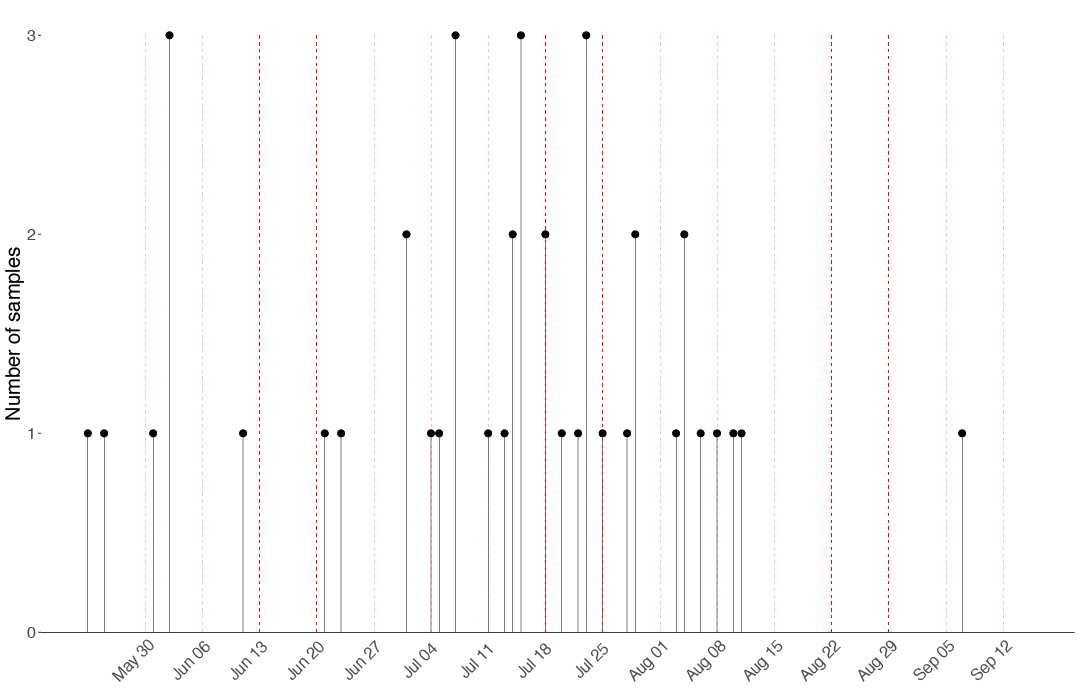

Supplement: Van der Roest et al. supplementary material [file S0950268825100587sup001.zip › S4_timeline.png]

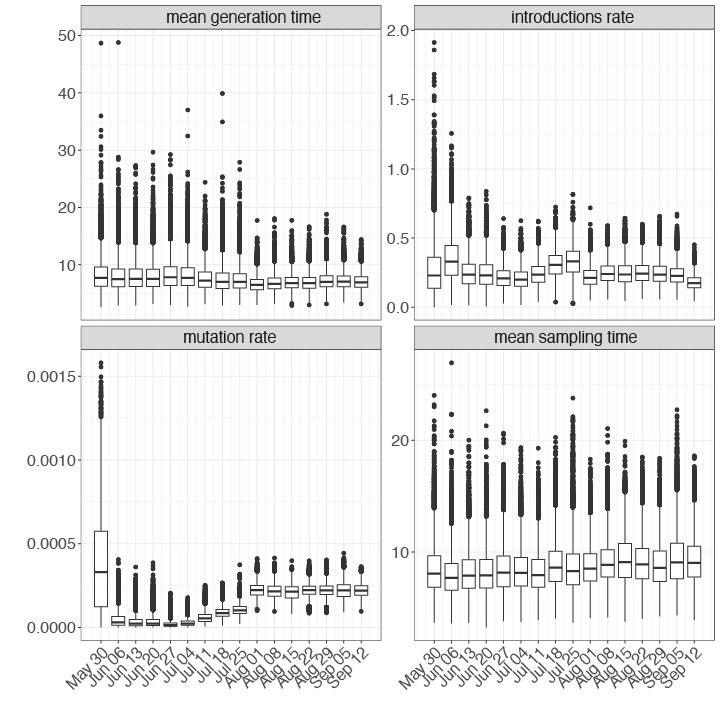

Supplement: Van der Roest et al. supplementary material [file S0950268825100587sup001.zip › S6_posteriors.png]
